# Alcian blue‐positive stromal phenotype in basal cell carcinoma is associated with progression on first‐line hedgehog inhibitors

**DOI:** 10.1002/2056-4538.70074

**Published:** 2026-01-16

**Authors:** Viola K DeTemple, Rudolf Stadler, Sabine Bredemeier, Sungyoung Chung, Katrin Schaper‐Gerhardt, Mareike Alter, Yenny Angela, Henner Stege, Ulrike Leiter, Jan Ohletz, Elisabeth Livingstone, Imke von Wasielewski, Jessica C Hassel, Julia Huynh, Christoffer Gebhardt, Claudia Pföhler, Ralf Gutzmer, Christina H Scheel

**Affiliations:** ^1^ Department of Dermatology, Johannes Wesling Medical Center Minden Ruhr‐University Bochum Bochum Germany; ^2^ Department of Dermatology University Hospital Mainz Mainz Germany; ^3^ Division of Dermatooncology, Department of Dermatology University Medical Center Tuebingen Germany; ^4^ Department of Dermatology Vivantes MVZ GmbH Berlin‐Spandau Germany; ^5^ Department of Dermatology University Hospital Essen Essen Germany; ^6^ Skin Cancer Research German Cancer Consortium (DKTK), Partner Site Essen/Duesseldorf Duesseldorf Germany; ^7^ Skin Cancer Center Hannover, Department of Dermatology and Allergy Hannover Medical School Hannover Germany; ^8^ Medical Faculty Heidelberg, Department of Dermatology and National Center for Tumour Diseases (NCT), NCT Heidelberg Heidelberg University Heidelberg Germany; ^9^ Department of Dermatology Charité – University Hospital Berlin Berlin Germany; ^10^ Skin Cancer Center, Department of Dermatology and Venereology University Medical Center Hamburg‐Eppendorf Hamburg Germany; ^11^ Department of Dermatology Saarland University Hospital and Saarland University Faculty of Medicine Homburg Germany; ^12^ Department of Dermatology St. Josef‐Hospital Bochum, Ruhr‐University Bochum Bochum Germany

**Keywords:** basal cell carcinoma, hedgehog inhibitors, vismodegib, sonidegib, tumor stroma, Alcian blue staining, glycosaminoglycans, desmoplasia, biomarkers, therapy resistance

## Abstract

Basal cell carcinoma (BCC) is the most frequent malignancy in fair‐skinned populations. Although curable in most cases, approximately 4% of patients develop locally advanced or metastatic disease (advBCC) requiring systemic therapy. Hedgehog pathway inhibitors (HHIs; vismodegib/sonidegib) constitute standard first‐line treatment, yet individual responses vary and no histopathological biomarker predicting therapeutic outcome exists. We conducted a retrospective, multicenter analysis of 70 BCCs encompassing clinically common and advanced stages. Routine hematoxylin and eosin and Alcian blue (AB; pH 2.5) staining was evaluated using a 17‐parameter, numerically encoded histopathology matrix spanning tumor morphology, stromal composition, and immune contexture. Data were mapped by unsupervised hierarchical clustering. Distinct AB staining patterns were observed: superficial and nodular BCCs typically exhibited an AB‐positive peritumoral border, whereas infiltrative and sclerosing subtypes displayed a diffuse AB‐positive desmoplastic stroma. The latter also correlated with advanced EADO clinical stages (correlation coefficients 0.46–0.48; *p* < 0.001). In a subset of 30 advBCCs obtained before or during HHI therapy, AB‐positive stroma was the only parameter independently associated with shorter progression‐free survival (multivariable hazard ratio = 23.8; 95% CI 4.02–141.3; *p* < 0.001). Established clinical or histological features failed to associate with outcome. Our findings identify diffuse AB‐positive stroma as a readily detectable feature of histologically aggressive BCC and as a candidate biomarker associated with progression under HHI treatment. Because AB staining is routine, inexpensive, and easily standardized, this phenotype represents an immediately implementable readout for prospective validation and a potential link between extracellular‐matrix remodeling and therapy resistance in BCC.

## Introduction

Basal cell carcinoma (BCC) is the most frequent malignancy in fair‐skinned populations and its incidence continues to rise worldwide [1]. In Germany, approximately 200 new cases per 100,000 inhabitants occur annually [2]. Surgical excision is curative in the majority of cases. However, about 4% of patients present with locally advanced, multifocal, or metastatic disease (advBCC), for which surgery is not feasible and individualized risk assessment strategies are lacking [3].

For these advanced cases, systemic therapies targeting the hedgehog (HH) signaling pathway, specifically the Smoothened inhibitors vismodegib and sonidegib, are recommended as first‐line treatment. In second‐line settings, immune checkpoint blockade with the programmed cell death‐1 inhibitor cemiplimab is available. Despite the central role of HH signaling in BCC pathogenesis, with >85% of tumors harboring HH pathway mutations [4], clinical responses remain heterogeneous. Objective response rates (ORRs) to HH inhibitors (HHIs) range from 48% to 61% [5], and second‐line cemiplimab achieves ORRs of only 24–31% [6]. These limitations have prompted growing interest in optimizing treatment sequences [7] and exploring neoadjuvant approaches [8]. Reliable biomarkers to guide patient selection and predict therapeutic response are urgently needed.

Histopathological subtype classification according to the 4th edition of the World Health Organization (WHO) classification of skin tumors provides important prognostic information and is included in current clinical guidelines [9, 10, 11]. Nodular (nodBCC) and superficial (supBCC) histological subtypes generally carry a low risk for recurrence, whereas infiltrative (infBCC) and sclerosing (sclBCC) subtypes are associated with aggressive local behavior. Histology currently informs surgical decision‐making and recurrence risk assessment but is not used to stratify patients for systemic therapy [12]. The most recent European consensus guidelines (EADO) even categorize BCCs by treatment difficulty rather than by histopathological risk [3]. Consequently, routine pathology remains an underutilized source of predictive information for systemic therapy outcomes.

A growing body of evidence highlights the tumor microenvironment, particularly the extracellular matrix (ECM) and stromal remodeling, as a critical determinant of tumor invasion, metastasis, and therapeutic resistance across cancer types. In BCC, however, the stromal compartment has received little attention as a biomarker source. To address this gap, we performed a retrospective, multicenter histopathological analysis of both clinically common and advanced BCCs, combining conventional hematoxylin and eosin (H&E) with Alcian blue staining at pH 2.5, which labels acidic glycoconjugates such as glycosaminoglycans and sialylated mucins. Using a multiparametric, numerically encoded evaluation matrix and unsupervised clustering, we systematically mapped histological heterogeneity and its clinical correlates.

This approach revealed a previously underrecognized stromal phenotype, diffuse Alcian blue positivity, that was highly enriched in aggressive infBCC and sclBCC subtypes. We hypothesized that this acidic glycoconjugate‐enriched stromal state reflects a distinct microenvironmental program that promotes tumor invasion and reduces therapeutic vulnerability. Here, we show that this histological feature, readily detectable in routine pathology, is strongly associated with disease progression under first‐line HHI therapy. Our findings position Alcian blue‐positive stroma as a promising candidate biomarker for treatment resistance and as a potential mechanistic link between stromal remodeling and drug response in BCC.

## Materials and methods

### Patients and samples

Formalin‐fixed paraffin‐embedded (FFPE) samples of 70 histologically confirmed BCC from 62 patients were included (Table 1 and supplementary material, Table S1). Ten skin‐cancer centers in Germany (Berlin‐Charité, Berlin‐Spandau, Essen, Hannover, Heidelberg, Homburg, Mainz, Minden, Tuebingen, Hamburg‐Eppendorf) contributed 43 clinically advanced BCC samples of 35 patients. Advanced BCC (advBCC) was defined as stages IIB‐IV according to the European Association of Dermato‐Oncology (EADO) classification [3]. Among these, 30 samples from 27 patients with advBCC were collected before or during systemic first line treatment with HHI (supplementary material, Table S2). Of these, 25 samples were obtained prior to treatment initiation and 5 shortly after therapy start; serial on‐treatment biopsies were not available. Additionally, 27 clinically common BCC samples of 27 patients were included. To reduce selection bias, consecutive BCC cases between November 15 and 22, 2023 were chosen from the histopathological data base of the skin‐cancer center in Minden.

**Table 1:** Patient characteristics according to clinical BCC stage [European consensus guidelines (EADO)]

Clinical BCC stage			All	Common BCC (stage I/IIA)	MultiBCC (stage IIB)	laBCC (stage III)	metBCC (stage IV)	*p*
*N* (patients)			62	27	11	20	4	
Sex	Male	*n* (%)	35 (56.5)	17 (62.9)	5 (45.5)	10 (50.0)	3 (75.0)	0.59*
	Female	*n* (%)	27 (43.5)	10 (37.1)	6 (54.5)	10 (50.0)	1 (25.0)	
Age at primary treatment	Median, years (range)		75 (31–94)	75 (55–94)	63 (31–87)	76.5 (46–88)	75.5 (43–82)	0.24^†^
	IQR		66.0–82.0	68.0–81.0	55.0–76.5	69.8–84.0	63.3–81.3	
Gorlin‐Goltz‐syndrome	Yes	*n* (%)	6 (9.7)	0 (0.0)	5 (45.5)	1 (5.0)	0 (0.0)	1.7e‐04*
	No	*n* (%)	56 (90.3)	27 (100)	6 (54.5)	19 (95.0)	4 (100)	
Received systemic treatment	Yes	*n* (%)	35 (56.5)	0 (0.0)	11 (100)	20 (100)	4 (100)	2.2e‐13*
	No	*n* (%)	27 (43.5)	27 (100)	0 (0.0)	0 (0.0)	0 (0.0)	
Previous BCC	Yes	*n* (%)	34 (54.8)	9 (33.3)	10 (90.9)	12 (60.0)	3 (75.0)	2.5e‐03*
	No	*n* (%)	25 (40.3)	18 (66.7)	1 (9.1)	6 (30.0)	0 (0.0)	
	NA	*n* (%)	3 (4.8)	0 (0.0)	0 (0.0)	2 (10.0)	1 (25.0)	
BCC relapse at same location	Yes	*n* (%)	11 (17.7)	0 (0.0)	0 (0.0)	8 (40.0)	3 (75.0)	1.5e‐09*
	No	*n* (%)	33 (53.2)	27 (100)	6 (54.5)	0 (0.0)	0 (0.0)	
	NA	*n* (%)	18 (29.0)	0 (0.0)	5 (45.5)	12 (60.0)	1 (25.0)	

BCC, basal cell carcinoma; IQR, interquartile range; laBCC, locally advanced BCC; metBCC, metastasized BCC; multiBCC, multiple BCCs; NA, not available; syst., systemic.

* Pearson's chi‐squared test.

^†^ Kruskal–Wallis rank sum test.

The following clinical data were collected per patient: sex; age at primary treatment (surgical excision for common BCC, initiation of HHI for advBCC); previous BCC; BCC relapse at the same location; metastasis (Table 1). For patients with advBCC, additional treatment‐related parameters were recorded: best response; progression status; treatment‐specific progression‐free survival (PFS); treatment duration; reason for treatment discontinuation; follow‐up time (supplementary material, Table S2).

The following tumor parameters were collected: localization; tumor thickness; tumor type; resection status; ulceration status; treatment naivety (supplementary material, Table S1).

The study was conducted in accordance with the Declaration of Helsinki and approved by the Ethics Committee of Ruhr University Bochum Site Bad Oeynhausen, Germany (protocol code 2021–811; date of approval: 8 June 2021). Written informed consent was obtained from all patients for inclusion in this study and for use of their anonymized clinical and histological data for research purposes.

### Histology staining

Sections of FFPE samples were cut at 5 μm thickness. After deparaffinization, slides were stained with hematoxylin (epredia, Portsmouth, NH, USA) and eosin (DiaPath, Martinengo, Italy) using an automated staining apparatus (Tissue‐Tek Prisma, Sakura, Torrance, CA, USA). For Alcian blue staining, deparaffinized sections were stained with Alcian blue solution pH 2.5 (BioGnost, Zagreb, Croatia) and Nuclear Fast Red counterstain according to the manufacturer's protocol. Slides were digitized using a Hamamatsu NanoZoomer slide scanner with the NDP.view2 software (Hamamatsu, Hamamatsu City, Japan).

### Evaluation matrix

**Figure 1:**
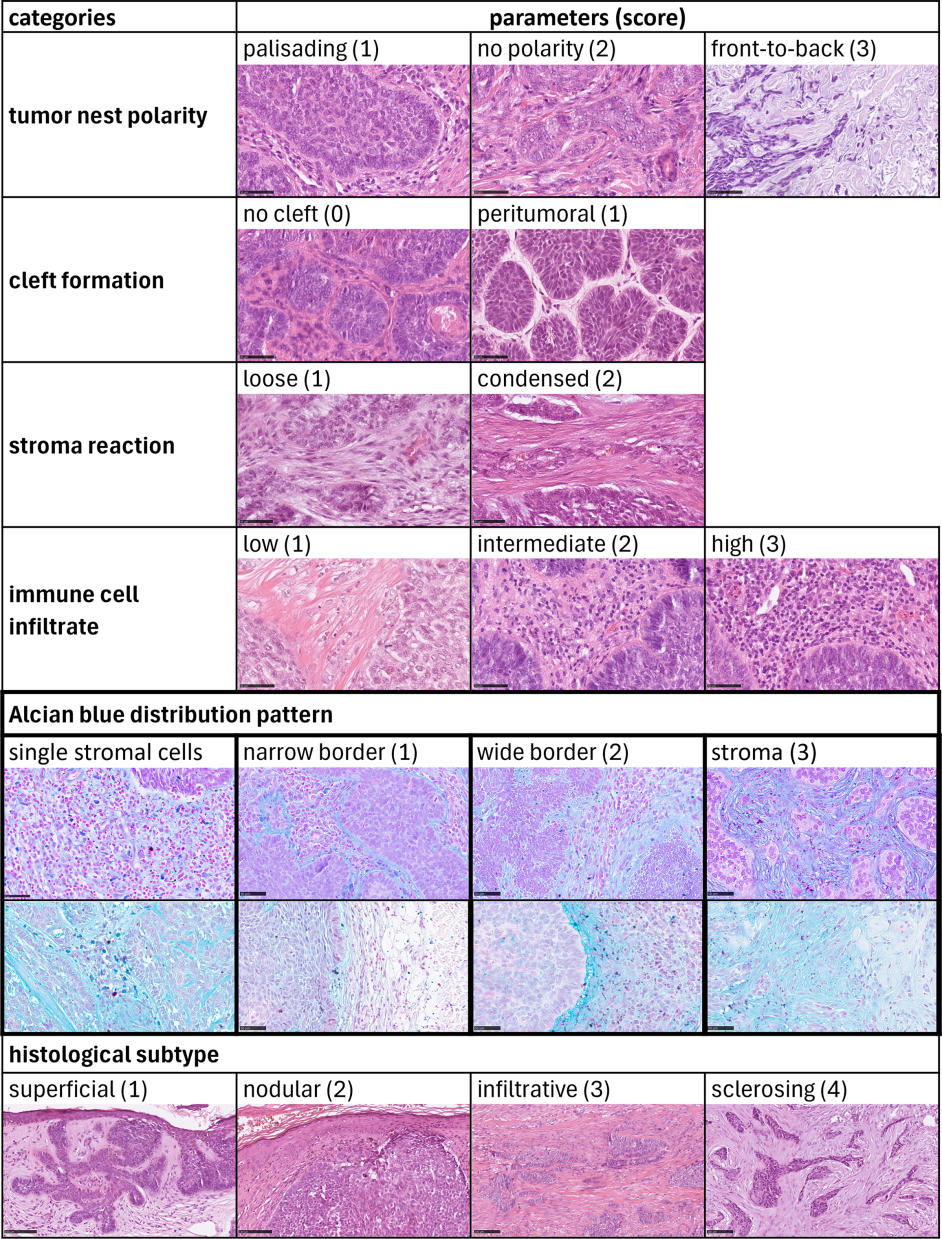
Evaluation matrix. Overview of the six histopathological categories chosen (rows) with corresponding parameters per category (columns). Scores for heatmap annotation and correlation analysis are provided in brackets per parameter. Representative histological images per parameter are shown: rows 1 to 4 and row 7 with H&E staining; rows 5 and 6 with Alcian blue staining pH 2.5. Scale bars represent 50 μm in rows 1 to 6 or 100 μm in row 7.

A predefined evaluation matrix comprised six histopathological categories with 2–4 parameters each (Figure 1): (1) tumor nest polarity (palisading, no polarity, front‐to‐back), (2) cleft formation (no clefting, peritumoral clefting), (3) stromal reaction (loose, condensed), (4) immune cell infiltrate (low, intermediate, high), (5) Alcian blue distribution pattern (narrow border (<median), wide border (>median), diffuse in the entire stroma, single positive stromal cells; supplementary material, Figure S1); (6) histological subtype according to the 4th edition of the WHO classification (superficial, nodular, infiltrative, sclerosing). Of note, none of the very rare BCC variants (e.g., basosquamous/metatypic carcinoma) were encountered in our material. As these entities fall outside the major WHO‐defined BCC subtypes and were not available for evaluation, they were excluded from the analysis and no conclusions can be made about their stromal or Alcian blue staining characteristics.

For each category, the dominant parameter was determined for correlation analysis and heatmap annotation. Intratumoral heterogeneity was quantified as the percentage of each parameter within a sample. Three investigators, blinded to clinical data and treatment outcomes, evaluated all slides. Scoring discrepancies were resolved by joint review, resulting in full consensus (interobserver agreement 100%; supplementary material, Table S3).

### Statistics

The date of surgery (common BCCs) or HHI initiation (advBCCs) served as the index date. Follow‐up period and OS were measured from the index date until death, last contact date, or end of observation period (July 2024), whichever occurred first. OS was omitted due to low occurrence of death within the observation period (*n* = 8, 10.67%). PFS under HHI was defined as the interval from HHI initiation to disease progression or death, whichever occurred first. Patients without progression or death were censored at the date of treatment change, last follow‐up, or data cut‐off. Best response and progression were determined by the respective skin cancer center, using clinical, radiological, and/or histological assessment. Descriptive statistics (medians, interquartile ranges, time intervals and percentual ratios) were calculated in Microsoft Excel (Microsoft 365, version 2419).

Group comparisons used Pearson's chi‐squared test (for categorical data) and Kruskal–Wallis rank sum test (for continuous data) and were performed using the stats‐package in R (version 4.4.1, R Project for Statistical Computing) [13]. For Kaplan–Meier analysis as well as uni‐ and multivariate analysis (Cox proportional hazards model), the packages survival (version 3.6‐4) [14] and survminer (version 0.4.9) [15] in R were used. Correlation matrices were generated with the package corrplot (version 0.95) [16] in R. The heatmap including unsupervised hierarchical clustering was created using the package ComplexHeatmap (version 2.20.0) [17]. Visualizations in R were supported by the packages circlize (version 0.4.16) [18], ggpubr (version 0.6.0) [19], and ggplot2 (version 3.5.1) [20]. Analyses were conducted on complete cases. Missing data per variable were <10% overall.

## Results

### Patient cohort and clinicopathological characteristics

A total of 70 BCC samples from 62 patients were analyzed (Table 1 and supplementary material, Table S1). Each sample was analyzed as an independent histological entity; no repeated‐measures correction was applied given limited overlap. Histologically, the cohort consisted of 11 superficial BCCs (supBCC; 15.7%), 34 nodular BCCs (nodBCC; 48.6%), 22 infiltrative BCCs (infBCC; 31.4%), and 3 sclerosing BCCs (sclBCC; 4.3%).

The clinically advanced sub‐cohort (advBCC) comprised 43 samples from 35 patients and included (1) high multiplicity BCC (multiBCC, EADO stage IIB; *n* = 13 samples from 11 patients), of which 5 patients were diagnosed with Gorlin–Goltz syndrome; (2) locally advanced, inoperable BCC (laBCC, EADO stage III; *n* = 25 samples from 20 patients); and (3) primary tumor samples from patients with metastatic disease (metBCC, EADO stage IV; *n* = 5 samples from 4 patients). Histological subtypes within the advBCC group included predominantly nodBCC (46.5%, *n* = 20), followed by infBCC (44.2%, *n* = 19), sclBCC (7.0%, *n* = 3), and supBCC (2.3%, *n* = 1).

To assess the predictive value of histopathological parameters for HHI response, 30 advBCC samples from 27 patients were collected prior to or during HH inhibition (supplementary material, Table S2). Of these, 25 biopsies were obtained before treatment initiation, while only 5 samples were taken shortly after the start of HHI therapy. True longitudinal on‐treatment sampling was therefore not available, and no conclusions can be drawn regarding dynamic morphological changes or alterations in Alcian blue‐positive stromal patterns during therapy.

Independent of histological subtype, treatment response rates were consistent with published data, with an ORR of 88.9% (complete response 22.2%; partial response 66.7%) and a median progression‐free survival (PFS) of 23.4 months under first‐line HHI therapy [5, 6]. ORR was higher than reported in larger phase II trials, likely reflecting referral bias in our cohort. This subset was supplemented by consecutively selected common BCC samples (EADO stages I/IIA; *n* = 27 samples from 27 patients) reflecting expected histological distributions: nodBCC (51.9%, *n* = 14), supBCC (37.0%, *n* = 10), and infBCC (11.1%, *n* = 3; supplementary material, Table S3) [21].

### Heatmap‐based analysis reveals histological clustering patterns

**Figure 2:**
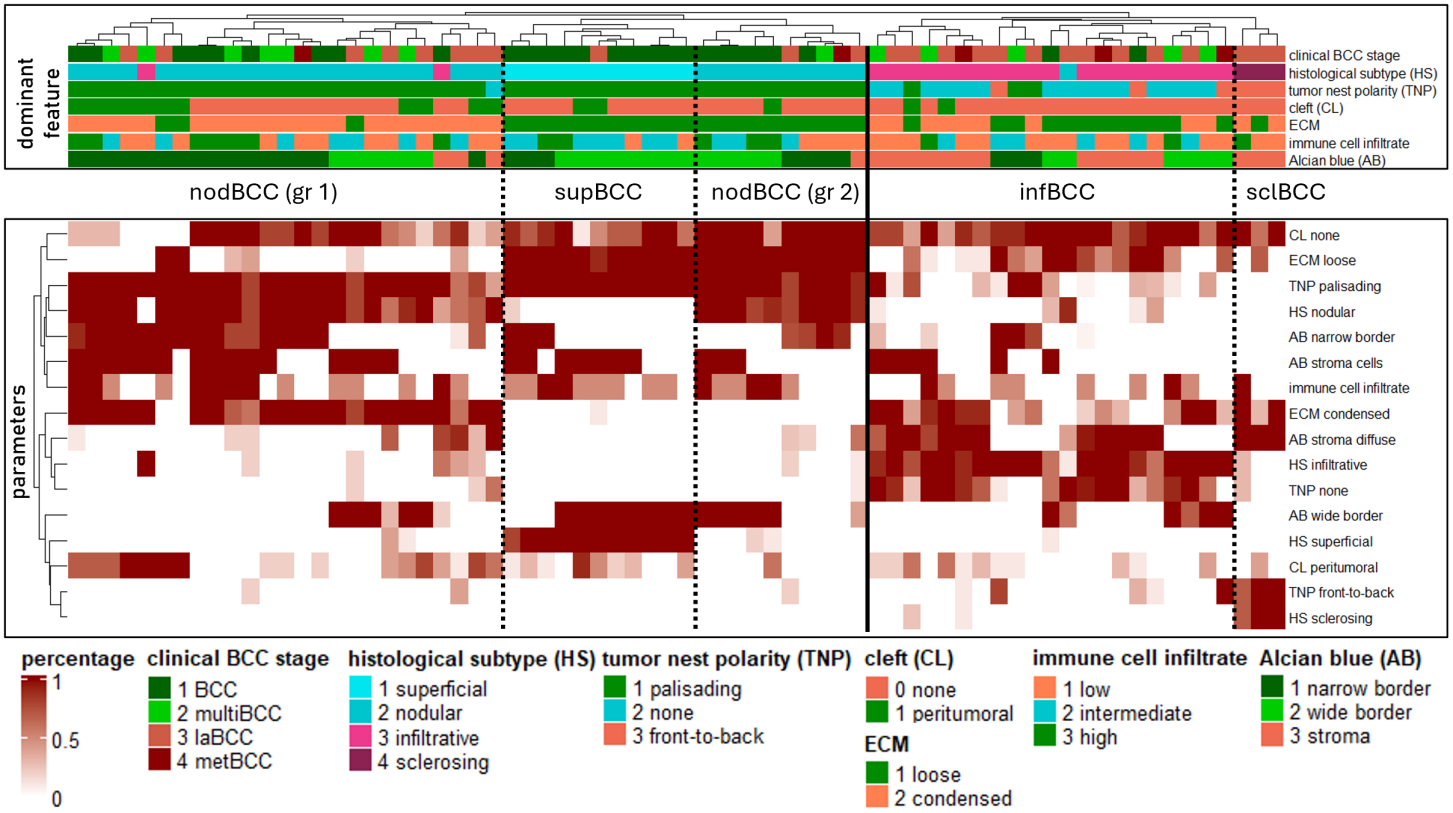
Prognostic value of histological parameters and Alcian blue distribution in BCC. Heatmap of parameters in percentage per category (AB, Alcian blue distribution; CL, cleft formation; ECM, extracellular matrix; HS, histological subtype; IC, immune cell infiltrate; TNP, tumor nest polarity), heat ranges between 0 and 1. Annotation according to dominant parameter. Unsupervised hierarchical clustering. The solid line signifies unsupervised separation between non‐aggressive (supBCC/nodBCC) and aggressive (infBCC/sclBCC) histological subtypes; dotted lines highlight further unsupervised clusters according to histological subtype.

To comprehensively assess histomorphological heterogeneity, we designed a 17‐parameter evaluation matrix encompassing six histopathological categories (Figure 1 and supplementary material, Table S3). Data derived from H&E‐ and Alcian blue‐stained FFPE sections were visualized as a heatmap, with color intensity indicating parameter frequency and annotation representing the dominant parameter per category. Unsupervised hierarchical clustering produced groups largely corresponding to conventional histological subtypes, validating the selection of parameters (Figure 2). Additionally, novel patterns emerged. Notably, histologically aggressive infBCCs and sclBCCs formed closely related clusters, whereas nodBCC and supBCC samples were more intermixed. This clustering pattern may reflect shared morphogenetic programs rather than discrete histotypes. Infiltrative BCCs mostly lacked peripheral palisading or displayed a front‐to‐back polarity, whereas non‐aggressive subtypes frequently exhibited basal‐apical polarity alongside peritumoral clefting.

Interestingly, nodBCCs split into two distinct subgroups. One, positioned between infBCC and supBCC clusters, was characterized by condensed stroma with densely eosinophilic extracellular fibers but without increased fibroblast content. The second nodBCC subgroup clustered closer to supBCC and exhibited loose stroma with sparse, faintly eosinophilic fibers. These findings suggest the existence of two nodBCC subtypes or, alternatively, a histological spectrum reflecting increasing expansive growth and stromal compression.

### Alcian blue reveals distinct stromal phenotypes across BCC subtypes

While H&E staining primarily refined classical subtype distinctions, Alcian blue staining uncovered additional, previously underappreciated stromal features (Figure 1). Non‐aggressive subtypes (supBCC and nodBCC) frequently displayed a narrow or wide Alcian blue‐positive peritumoral border neatly following the contours of each tumor nest (supplementary material, Figure S1D/E). The border width varied between 2 μm and 119.8 μm, with a median of 16.2 μm (supplementary material, Table S3), independent of cleft formation. Employing the median as cut‐off, we divided this group into narrow border (2–16.2 μm border width) and wide border (16.3–119.8 μm border width). Occasional Alcian blue‐positive stromal cells with fibroblast‐like morphology were also observed in these cases.

In contrast, the majority of infBCCs and all sclBCCs demonstrated a second pattern, in which tumor nests were embedded within a uniformly diffuse Alcian blue‐positive stroma, rich in fibroblastoid cells and sparse immune infiltrates; a pattern comparable with islands within a deep blue ocean (supplementary material, Figure S1B/C). This diffuse blue stroma incorporated all tumor nests and tapered off to the periphery, at times as a clear border.

This striking difference in staining pattern indicates a fundamental stromal divergence between aggressive and non‐aggressive subtypes.

### Correlation between histopathological parameters and clinical stage

**Figure 3:**
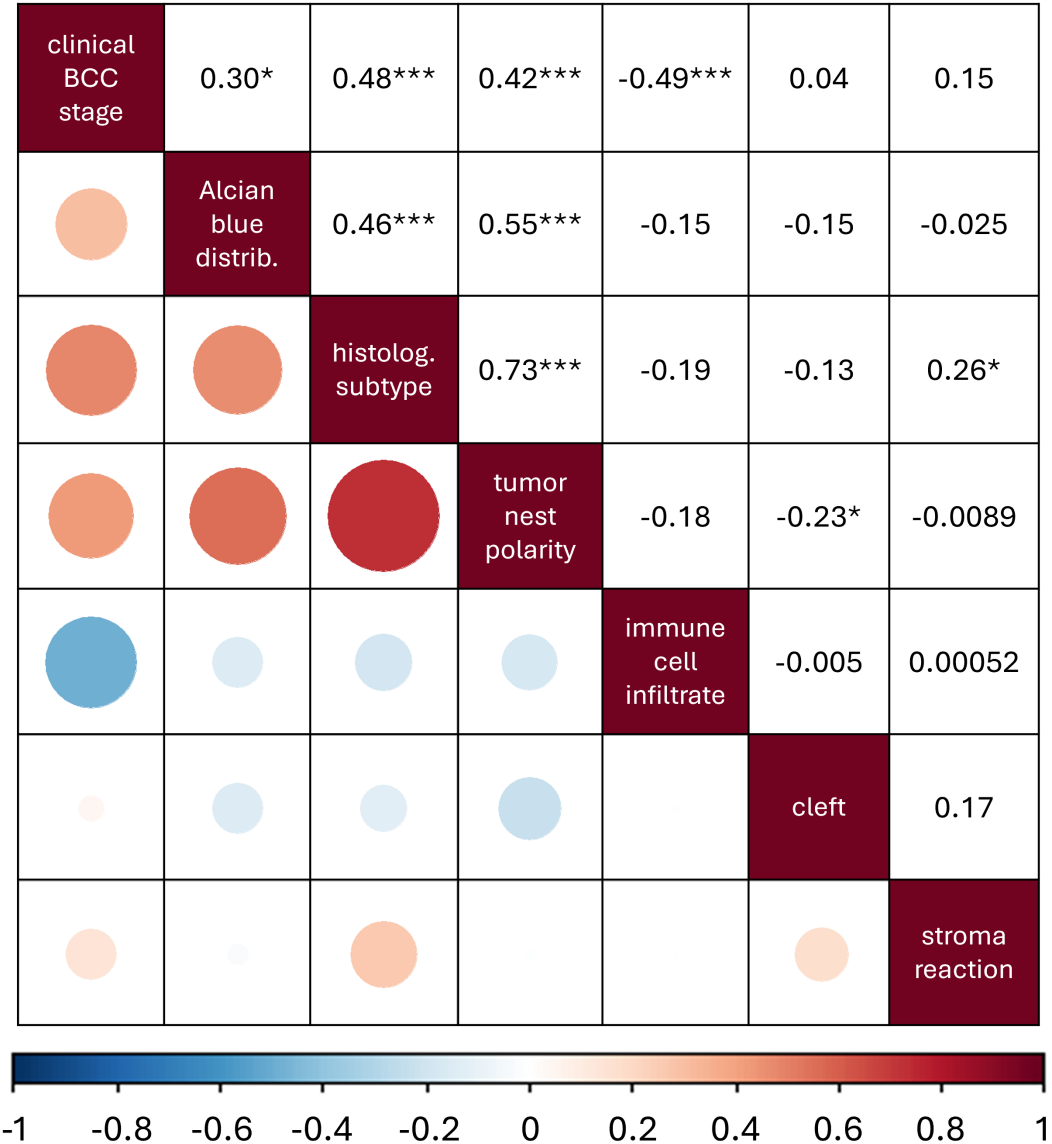
Correlation matrix comparing clinical stage and analyzed histological parameters. Dot size and color in the lower left as well numbers in the upper right represent correlation coefficients between −1 and 1. *p < 0.05, **p < 0.01, ***p < 0.001.

We next correlated histopathological parameters with EADO clinical stages (common BCC: I/IIA; advanced BCC: IIB–IV). As expected, the histological subtype strongly correlated with tumor nest polarity [Pearson correlation coefficient (CC) = 0.73, *p* < 0.001; Figure 3]. Importantly, Alcian blue positivity correlated with both histological subtype (CC = 0.46, *p* < 0.001) and nest polarity (CC = 0.55, *p* < 0.001), identifying diffuse Alcian blue‐positive stroma as a shared feature of aggressive subtypes.

Additional correlations linked histologically aggressive subtype, loss of nest polarity, and Alcian blue‐positive stroma with advanced clinical stage (CC = 0.48, *p* < 0.001; CC = 0.42, *p* < 0.001; CC = 0.30, *p* < 0.05, respectively). Conversely, immune cell infiltration negatively correlated with advanced stage (CC = −0.49, *p* < 0.001). All metastatic samples (*n* = 5) showed low immune cell infiltration, suggesting that immune evasion may contribute to metastatic progression. Together, these results support integrating histopathological features, including stromal phenotype, into staging systems for more refined risk assessment.

### Alcian blue‐positive stroma predicts poor response to HHI therapy

**Figure 4:**
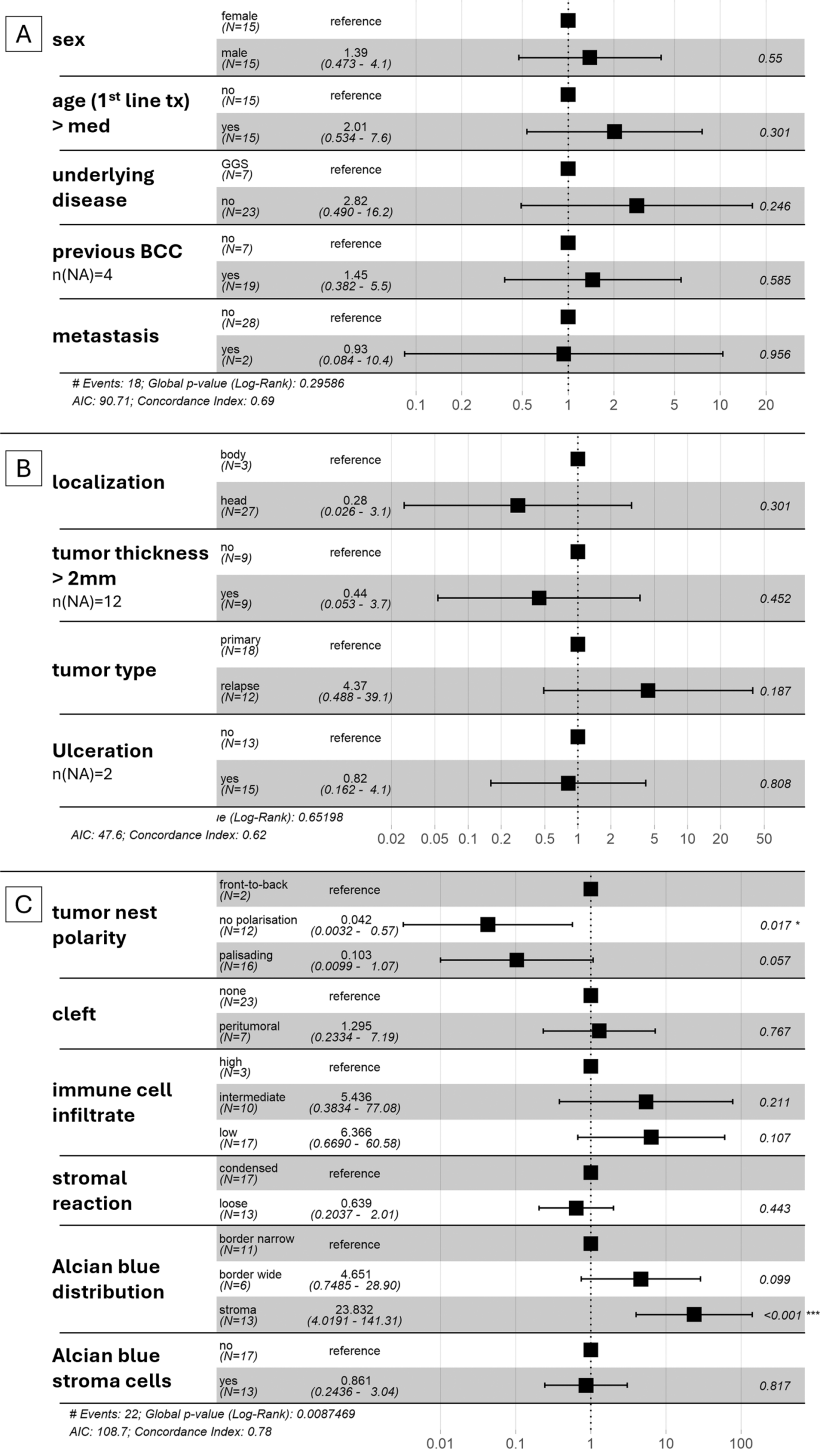
Predictive power of histopathological parameters and distribution of Alcian blue stain in BCC. Forrest plot (*n* = 30) of multivariate Cox regression of progression‐free survival following HHI treatment initiation with (A) clinical parameters, (B) conventional tumor parameters, and (C) analyzed histological parameters including Alcian blue stain distribution. Hazard ratio is given in numbers next to the parameters on the left and is presented as squares in the graph; whiskers represent hazard ratio range (also given in brackets). Vertical dotted line marks a hazard ratio of 1. Numbers on the right give *p* values (*p < 0.05, ***p < 0.001).

We next evaluated the predictive potential of histopathological features for response to HHI therapy in a subset of 30 BCC samples from 27 patients (supplementary material, Table S4). In multivariate Cox regression, Alcian blue‐positive stroma was the only parameter significantly associated with reduced PFS [HR = 23.832, CI (4.02–141.3), *p* < 0.001; Figure 4C]. Neither established clinical variables (Figure 4A) nor conventional tumor characteristics (Figure 4B) predicted PFS.

**Figure 5:**
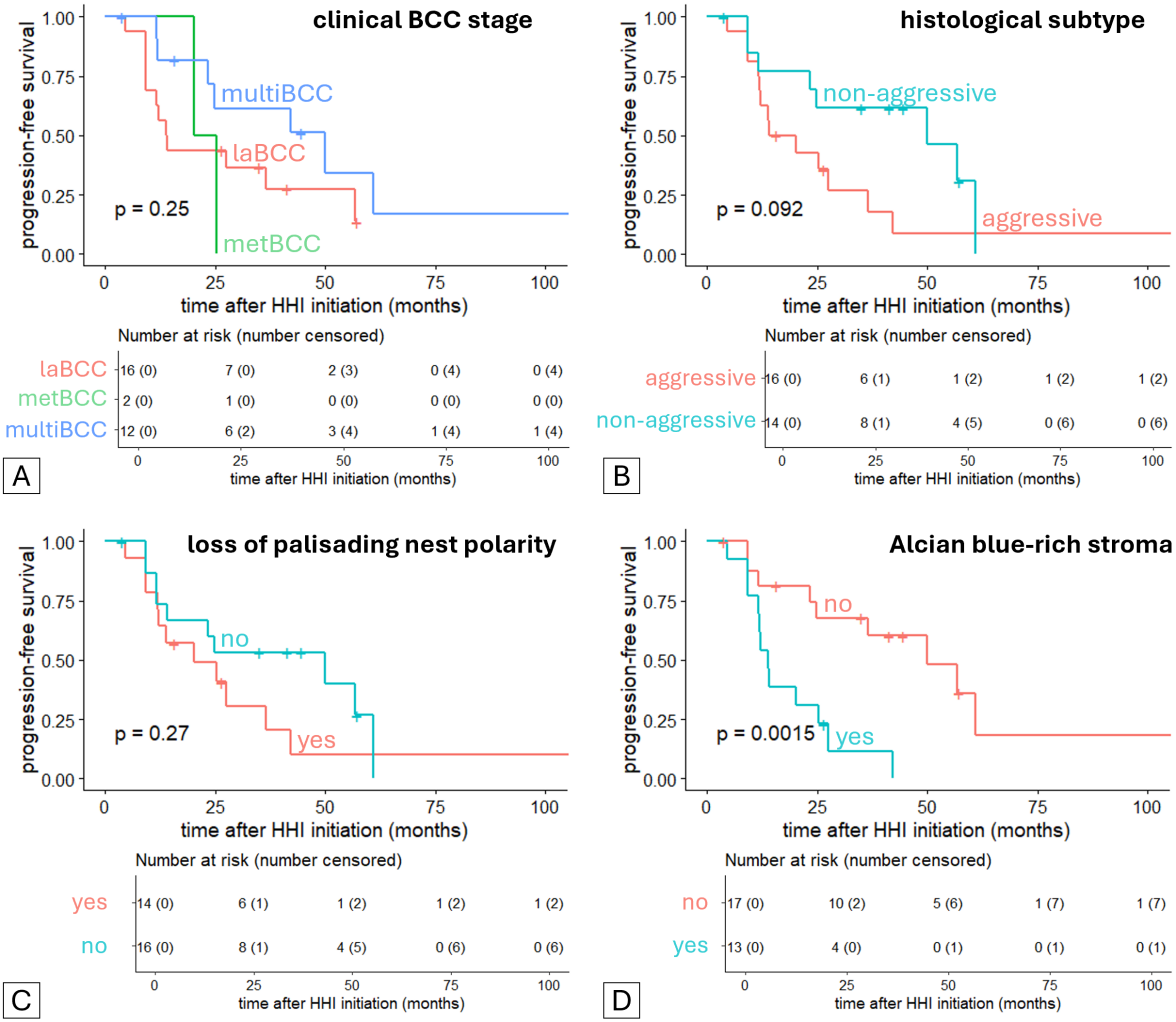
Survival analysis of clinical and pathological parameters in BCC. Kaplan–Meier regression (*n* = 30) of progression‐free survival following HHI treatment initiation for the following factors: (A) clinical BCC stage, (B) histological subtype, (C) palisading nest polarity, and (D) Alcian blue staining distribution in stroma.

Kaplan–Meier analysis confirmed this finding: clinical stage (Figure 5A), histological subtype (Figure 5B), and loss of palisading polarity (Figure 5C) showed no significant predictive value, whereas Alcian blue‐positive stroma was strongly associated with progression under HHI treatment (*p* = 0.0015; Figure 5D). Collectively, these data identify Alcian blue‐positive stroma as a strong candidate independent biomarker for HHI resistance, closely linked to histological aggressiveness and advanced disease stage. If prospectively validated, this feature could inform early therapeutic decisions, such as prioritizing combination regimens in HHI‐resistant stroma‐rich tumors.

## Discussion

In this study, we identify Alcian blue‐positive stroma as a robust histological correlate of aggressive BCC subtypes that is strongly associated with resistance to HHI therapy. Furthermore, our multiparametric, numerically encoded histopathological approach effectively captured clinically relevant tumor features and revealed novel stromal patterns with prognostic and predictive implications.

Our findings complement and extend previous transcriptomic evidence showing that histologically aggressive BCCs (infBCC and sclBCC) are molecularly distinct from nodular subtypes [22]. While most studies on HHI resistance have focused on tumor‐intrinsic mechanisms, including canonical mutations in *SMO*, *SUFU*, or *GLI2* that reactivate HH signaling, these alterations alone do not fully explain clinical variability in treatment response [23, 24, 25]. Indeed, baseline GLI1 expression did not correlate with sonidegib efficacy in the BOLT trial, limiting its value as a predictive biomarker [24]. Moreover, non‐canonical resistance mechanisms involving microenvironmental factors, such as AP‐1 or TGF‐β signaling or cytoskeletal pathways mediated by Rho/MKL1, are increasingly recognized as critical contributors [26, 27].

Our study adds to this evolving picture by implicating the tumor stroma as a potential determinant of HHI response. Alcian blue staining has traditionally been used to detect acidic glycoconjugates, including mucins and sulfated polysaccharides [28]. This includes acidic glycosaminoglycans, sulfated mucopolysaccharides, and sialomucins [29, 30, 31]. Notably, proteoglycans such as glypicans and syndecans, and their glycosaminoglycan chains, are essential for HH ligand distribution and reception [32, 33]. Thus, an acidic glycoconjugate‐rich stromal environment may directly modulate HH pathway activity and influence therapeutic response. Biochemical analyses dissecting the composition of the Alcian blue‐positive ECM will be crucial to elucidate its mechanistic contribution to resistance.

The origin of this stromal phenotype remains to be clarified. Its association with a fibroblast‐rich desmoplastic matrix suggests that stromal fibroblasts produce the acidic components visualized by Alcian blue. In cases with a narrow peritumoral border, Alcian blue‐positive fibroblast‐like cells were observed at the tumor‐stroma interface, potentially representing precursors to the fibroblast expansion seen in diffusely positive stroma. Dermal papilla fibroblasts, which secrete hyaluronic acid and other glycosaminoglycans that regulate HH signaling [34], stain intensely with Alcian blue. Given known parallels between BCC and embryonic hair follicle morphogenesis, including expression of epithelial cell adhesion molecule [35], raises the possibility that BCC‐associated fibroblasts undergo reprogramming toward a dermal papilla‐like state [36].

To our knowledge, diffuse Alcian blue‐positive stroma has not previously been described as a histopathological feature of BCC. Its detection using standard staining protocols enables immediate integration into routine pathology workflows, underscoring the translational potential of this biomarker. We acknowledge that the cohort size is limited, reflecting the rarity of advanced BCC, and that larger multicenter studies will be required to validate the predictive value of Alcian blue‐positive stromal patterns. Future studies should also determine whether Alcian blue‐positive stroma contributes to HH pathway modulation and further assess its potential relevance for patient stratification and therapy selection.

## Author contributions statement

VKD and CHS conceived, designed and conducted the study and wrote the manuscript. VKD compiled patient data, conducted statistical analysis and visualized the data. Histopathological analysis was done by VKD, RS and CHS. SB conducted histological staining; SC contributed to data curation. Advanced BCC cases were contributed by MA, YA, HS, UL, JO, EL, IvW, JCH, JH, CG and CP. All authors critically reviewed and approved the manuscript.

## Supporting information


**Figure S1.** Alcian blue staining patterns
**Table S1.** Conventional tumor parameters according to clinical BCC stage [European consensus guidelines (EADO)]
**Table S2.** Subcohort of patients with samples taken before or during hedgehog inhibition
**Table S3.** Analyzed histological tumor parameters according to clinical BCC stage [European consensus guidelines (EADO)]
**Table S4.** Univariate Cox proportional regression (progression‐free survival following HHI treatment initiation)

## Data Availability

All data supporting the findings of this study are available within the article and its supplementary materials. Detailed descriptions of the analysis workflow and algorithms are provided in the Materials and Methods, including direct links to the publicly accessible repositories hosting the analysis scripts and visualization pipelines (R). De‐identified clinical and histopathological datasets, together with the exact versions of the analysis scripts used for data encoding, clustering, correlation analysis, and survival modeling, are available from the corresponding author upon reasonable request.

## References

[1] Kauvar ANB , Cronin T , Roenigk R , *et al*. Consensus for nonmelanoma skin cancer treatment. Dermatol Surg 2015; 41: 550–571. 25868035 10.1097/DSS.0000000000000296

[2] Krensel M , Petersen J , Mohr P , *et al*. Estimating prevalence and incidence of skin cancer in Germany. J Dtsch Dermatol Ges 2019; 17: 1239–1249. 10.1111/ddg.14002 31885171

[3] Peris K , Fargnoli MC , Kaufmann R , *et al*. European consensus‐based interdisciplinary guideline for diagnosis and treatment of basal cell carcinoma – update 2023. Eur J Cancer 2023; 192: 113254. 37604067 10.1016/j.ejca.2023.113254

[4] Bonilla X , Parmentier L , King B , *et al*. Genomic analysis identifies new drivers and progression pathways in skin basal cell carcinoma. Nat Genet 2016; 48: 398–406. 26950094 10.1038/ng.3525

[5] Dummer R , Ascierto PA , Basset‐Seguin N , *et al*. Sonidegib and vismodegib in the treatment of patients with locally advanced basal cell carcinoma: a joint expert opinion. J Eur Acad Dermatol Venereol 2020; 34: 1944–1956. 31990414 10.1111/jdv.16230

[6] Stratigos AJ , Sekulic A , Peris K , *et al*. Cemiplimab in locally advanced basal cell carcinoma after hedgehog inhibitor therapy: an open‐label, multi‐centre, single‐arm, phase 2 trial. Lancet Oncol 2021; 22: 848–857. 34000246 10.1016/S1470-2045(21)00126-1

[7] DeTemple VK , Hassel JC , Sachse MM , *et al*. Reinduction of hedgehog inhibitors after checkpoint inhibition in advanced basal cell carcinoma: a series of 12 patients. Cancer 2022; 14: 5469. 10.3390/cancers14215469 PMC9658899 36358887

[8] Hoellwerth M , Brandlmaier M , Koelblinger P . Therapeutic approaches for advanced basal cell carcinoma: a comprehensive review. Cancer 2024; 17: 68. 10.3390/cancers17010068 PMC11718879 39796697

[9] Fernández‐Figueras MT , Malvehi J , Tschandl P , *et al*. Position paper on a simplified histopathological classification of basal cell carcinoma: results of the European Consensus Project. J Eur Acad Dermatol Venereol 2022; 36: 351–359. 34931722 10.1111/jdv.17849

[10] Dixon AY , Lee SH , McGregor DH . Histologic evolution of basal cell carcinoma recurrence. Am J Dermatopathol 1991; 13: 241–247. 1867354 10.1097/00000372-199106000-00005

[11] Lim GF‐S , Perez OA , Zitelli JA , *et al*. Correlation of basal cell carcinoma subtype with histologically confirmed subclinical extension during Mohs micrographic surgery: a prospective multicenter study. J Am Acad Dermatol 2022; 86: 1309–1317. 35231546 10.1016/j.jaad.2022.02.037

[12] Schmults CD , Blitzblau R , Aasi SZ , *et al*. Basal cell skin cancer, version 2.2024, NCCN clinical practice guidelines in oncology. J Natl Compr Canc Netw 2023; 21: 1181–1203. 37935106 10.6004/jnccn.2023.0056

[13] R Core Team . R: A Language and Environment for Statistical Computing. R Foundation for Statistical Computing: Vienna, 2024. [Accessed 6 August 2024]. Available from: https://www.R-project.org/.

[14] Therneau T . A Package for Survival Analysis in R. R Package Version 3.6‐4 2024. [Accessed 18 November 2024]. Available from: https://CRAN.R-project.org/package=survival.

[15] Kassambara A , Kosinski M , Biecek P . survminer: Drawing Survival Curves Using ‘ggplot2’, R Package Version 0.4.9 2021. [Accessed 18 November 2024]. Available from: https://CRAN.R-project.org/package=survminer.

[16] Wei T , Simko V . R Package ‘corrplot’: Visualization of a Correlation Matrix (Version 0.95) 2024. [Accessed 19 November 2024]. Available from: https://github.com/taiyun/corrplot.

[17] Gu Z . Complex Heatmap Visualization. iMeta 2022; 1: e43. 38868715 10.1002/imt2.43 PMC10989952

[18] Gu Z , Gu Z , Gu L , *et al*. circlize Implements and Enhances Circular Visualization in R. Bioinformatics. 2014; 30: 2811–2812. 24930139 10.1093/bioinformatics/btu393

[19] Kassambara A . ggpubr: ‘ggplot2’ Based Publication Ready Plots. R Package Version 0.6.0 2023. [Accessed 19 November 2024]. Available from: https://CRAN.R-project.org/package=ggpubr.

[20] Wickham H . ggplot2: Elegant Graphics for Data Analysis. Springer‐Verlag: New York, 2016.

[21] Scrivener Y , Grosshans E , Cribier B . Variations of basal cell carcinomas according to gender, age, location and histopathological subtype. Br J Dermatol 2002; 147: 41–47. 12100183 10.1046/j.1365-2133.2002.04804.x

[22] Villani R , Murigneux V , Alexis J , *et al*. Subtype‐specific analyses reveal infiltrative basal cell carcinomas are highly interactive with their environment. J Invest Dermatol 2021; 141: 2380–2390. 33865912 10.1016/j.jid.2021.02.760

[23] Nguyen NM , Cho J . Hedgehog pathway inhibitors as targeted cancer therapy and strategies to overcome drug resistance. Int J Mol Sci 2022; 23: 1733. 35163655 10.3390/ijms23031733 PMC8835893

[24] Migden MR , Guminski A , Gutzmer R , *et al*. Treatment with two different doses of sonidegib in patients with locally advanced or metastatic basal cell carcinoma (BOLT): a multicentre, randomised, double‐blind phase 2 trial. Lancet Oncol 2015; 16: 716–728. 25981810 10.1016/S1470-2045(15)70100-2

[25] Doan HQ , Chen L , Nawas Z , *et al*. Switching hedgehog inhibitors and other strategies to address resistance when treating advanced basal cell carcinoma. Oncotarget 2021; 12: 2089–2100. 34611482 10.18632/oncotarget.28080 PMC8487719

[26] Whitson RJ , Lee A , Urman NM , *et al*. Noncanonical hedgehog pathway activation through SRF–MKL1 promotes drug resistance in basal cell carcinomas. Nat Med 2018; 24: 271–281. 29400712 10.1038/nm.4476 PMC5839965

[27] Yao CD , Haensel D , Gaddam S , *et al*. AP‐1 and TGFß cooperativity drives non‐canonical hedgehog signaling in resistant basal cell carcinoma. Nat Commun 2020; 11: 5079. 33033234 10.1038/s41467-020-18762-5 PMC7546632

[28] Bjornsson S . Simultaneous preparation and quantitation of proteoglycans by precipitation with Alcian blue. Anal Biochem 1993; 210: 282–291. 8512063 10.1006/abio.1993.1197

[29] Reynolds IS , Fichtner M , McNamara DA , *et al*. Mucin glycoproteins block apoptosis; promote invasion, proliferation, and migration; and cause chemoresistance through diverse pathways in epithelial cancers. Cancer Metastasis Rev 2019; 38: 237–257. 30680581 10.1007/s10555-019-09781-w

[30] Wi D‐H , Cha J‐H , Jung Y‐S . Mucin in cancer: a stealth cloak for cancer cells. BMB Rep 2021; 54: 344–355. 34154702 10.5483/BMBRep.2021.54.7.064 PMC8328826

[31] Jonckheere N , Vincent A , Neve B , *et al*. Mucin expression, epigenetic regulation and patient survival: a toolkit of prognostic biomarkers in epithelial cancers. Biochim Biophys Acta 2021; 1876: 188538. 10.1016/j.bbcan.2021.188538 33862149

[32] Ortmann C , Pickhinke U , Exner S , *et al*. Sonic hedgehog processing and release are regulated by glypican heparan sulfate proteoglycans. J Cell Sci 2015; 128: 2374–2385. 25967551 10.1242/jcs.170670

[33] Whalen DM , Malinauskas T , Gilbert RJC , *et al*. Structural insights into proteoglycan‐shaped hedgehog signaling. Proc Natl Acad Sci 2013; 110: 16420–16425. 24062467 10.1073/pnas.1310097110 PMC3799379

[34] Steinmann S , Guillet C , Cheng PF , *et al*. Identifying the potential origin of mucin in primary cutaneous mucinoses – a retrospective study and analysis using histopathology and multiplex fluorescence staining. J Eur Acad Dermatol Venereol 2023; 37: 1302–1310. 36807595 10.1111/jdv.18992

[35] Sellheyer K , Krahl D . Basal cell (trichoblastic) carcinoma common expression pattern for epithelial cell adhesion molecule links basal cell carcinoma to early follicular embryogenesis, secondary hair germ, and outer root sheath of the vellus hair follicle: a clue to the adnexal nature of basal cell carcinoma? J Am Acad Dermatol 2008; 58: 158–167. 18158927 10.1016/j.jaad.2007.07.008

[36] Youssef KK , Lapouge G , Bouvrée K , *et al*. Adult interfollicular tumour‐initiating cells are reprogrammed into an embryonic hair follicle progenitor‐like fate during basal cell carcinoma initiation. Nat Cell Biol 2012; 14: 1282–1294. 23178882 10.1038/ncb2628

