# Supplementary material for: Alcian blue‐positive stromal phenotype in basal cell carcinoma is associated with progression on first‐line hedgehog inhibitors
Source: J Pathol Clin Res. 2026 Jan 16;12(1):e70074. doi: 10.1002/2056-4538.70074 (PMC12810521; doi:10.1002/2056-4538.70074)
Supplement: Supplementary file 1 — Figure S1. Alcian blue staining patterns Table S1. Conventional tumor parameters according to clinical BCC stage [European consensus guidelines (EADO)] Table S2. Subcohort of patients with samples taken before or during hedgehog inhibition Table S3. Analyzed histological tumor parameters according to clinical BCC stage [European consensus guidelines (EADO)] Table S4. Univariate Cox proportional regression (progression‐free survival following HHI treatment initiation) [file CJP2-12-e70074-s001.pdf]

Alcian blue-positive stromal phenotype in basal cell carcinoma is associated with progression on first-line hedgehog inhibitors

VK DeTemple *et al. J Pathol Clin Res* https://doi.org/10.1002/2056-4538.70074

Supplementary Figure S1

Supplementary Tables S1–S4

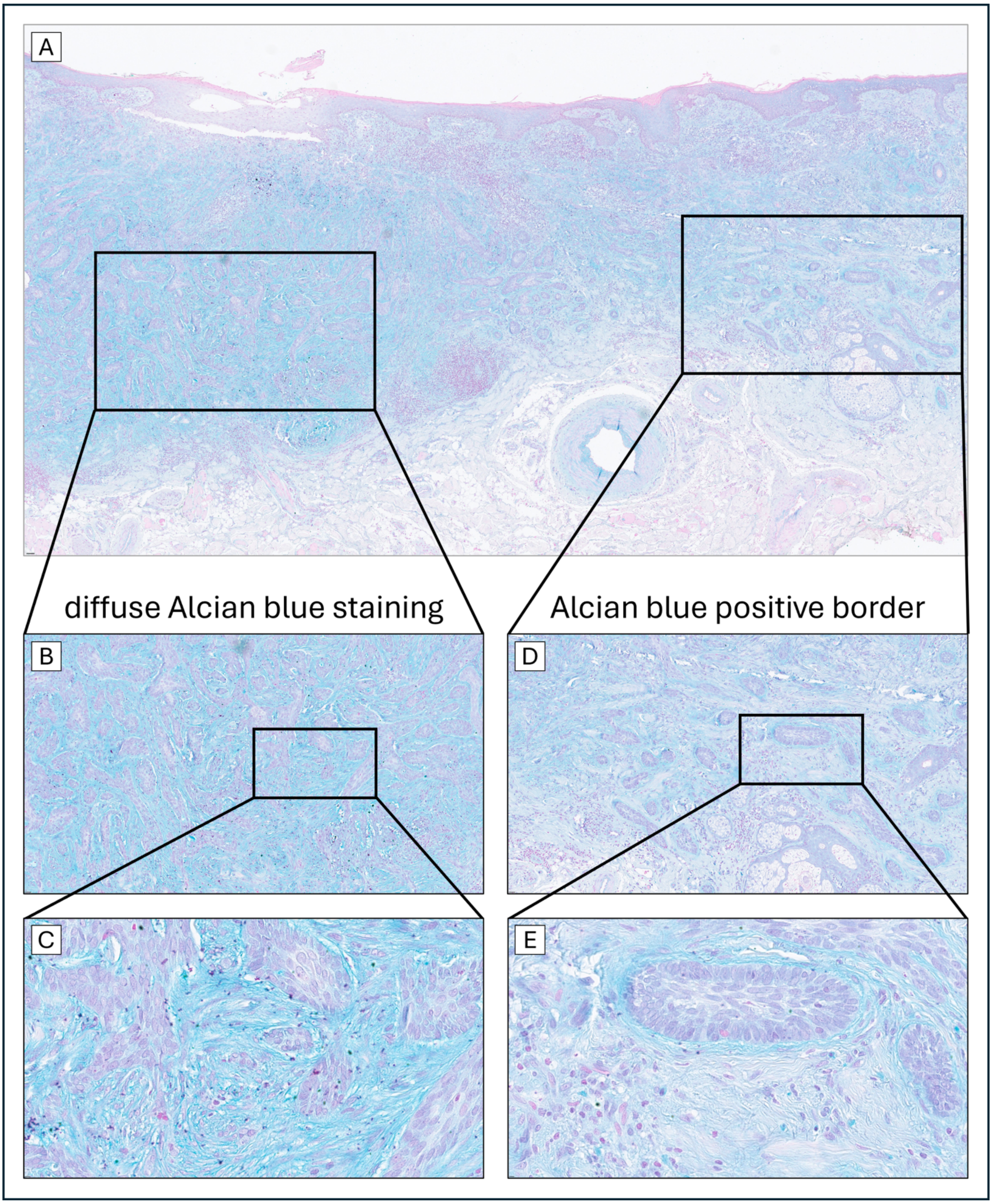

Figure S1. Alcian blue staining patterns. Representative image of a basal cell carcinoma demonstrating both a diffuse stromal pattern (A left, B, C) as well as an Alcian blue positive peritumoral border (A right, D, E) in different magnifications.

Table S1. Conventional tumor parameters according to clinical BCC stage [European consensus guidelines (EADO)]

BCC group			All	Common BCC (st. I/IIA)	MultiBCC (st. IIB)	laBCC (st. III)	metBCC (st. IV)	*p*
n (samples)			70	27	13	25	5	
Tumor localization	Head	*n* (%)	48 (68.6)	12 (44.4)	11 (84.6)	24 (96.0)	1 (20.0)	3.9e-05[a]
	Body	*n* (%)	22 (31.4)	15 (55.6)	2 (15.4)	1 (4.0)	4 (80.0)	
Tumor thickness	Median, mm (range)		1.6 (0.4–5.6)	1.2 (0.4–5.5)	1.6 (1.2–5.6)	2.2 (1.3–4.5)	4.0 (3.8–4.2)	0.0084[b]
	IQR		1.1–2.2	0.7–1.9	1.5–1.8	2.0–2.6	3.9–4.1	
Tumor thickness >2 mm	Yes	*n* (%)	16 (22.9)	6 (22.2)	1 (7.7)	7 (28.0)	2 (40.0)	0.0016[a]
	No	*n* (%)	31 (44.3)	21 (77.8)	8 (61.5)	2 (9.0)	0 (0.0)	
	NA	*n* (%)	23 (32.9)	0 (0.0)	4 (30.8)	16 (64.0)	3 (60.0)	
Tumor type	Primary	*n* (%)	45 (64.3)	26 (96.3)	12 (92.3)	6 (24.0)	1 (20.0)	2.3e-08[a]
	Relapse	*n* (%)	25 (35.7)	1 (3.7)	1 (7.7)	19 (76.0)	4 (80.0)	
Resection status	Biopsy	*n* (%)	13 (18.6)	1 (3.7)	2 (15.4)	9 (36.0)	1 (20.0)	3.2e-06[a]
	R1	*n* (%)	13 (18.6)	2 (7.4)	5 (38.5)	5 (20.0)	1 (20.0)	
	R0	*n* (%)	29 (41.4)	24 (88.9)	4 (30.8)	0 (0.0)	1 (20.0)	
	NA	*n* (%)	15 (21.4)	0 (0.0)	2 (15.4)	11 (44.0)	2 (40.0)	
Ulceration	Yes	*n* (%)	35 (50.0)	13 (48.2)	6 (46.2)	14 (56.0)	2 (40.0)	0.5245[a]
	No	*n* (%)	29 (41.4)	14 (52.9)	7 (53.8)	7 (28.0)	1 (20.0)	
	NA	*n* (%)	6 (8.6)	0 (0.0)	0 (0.0)	4 (16.0)	2 (40.0)	
Treatment naive sample	Yes	*n* (%)	52 (74.3)	27 (100)	10 (76.9)	13 (52.0)	2 (40.0)	0.0003[a]
	No	*n* (%)	18 (25.7)	0 (0.0)	3 (23.1)	12 (48.0)	3 (60.0)	

[a]Pearson's chi-squared test; [b]Kruskal–Wallis rank sum test.

BCC – basal cell carcinoma; multiBCC – multiple BCCs; laBCC – locally advanced BCC; metBCC – metastasized BCC; IQR – interquartile range; NA – not available; R1 – histologically incomplete resection; R0 – histologically complete resection.

Table S2. Subcohort of patients with samples taken before or during hedgehog inhibition

Patients with samples taken before or during 1st line hedgehog inhibition (HHI)							
Clinical data				Histological data			
n (patients)			27	*n* (samples)			30
Sex	Male	*n* (%)	14 (51.9)	Tumor localization	Head	*n* (%)	27 (90.0)
	Female	*n* (%)	13 (48.1)		Body	*n* (%)	3 (10.0)
Age at HHI initiation	Median, years (range)		76 (31–88)	Tumor thickness	Median, mm (range)		1.9 (1.2–5.6)
	IQR		62.5–84.0		IQR		1.5–2.6
Clinical BCC stage	MultiBCC	*n* (%)	12 (40.0)	Dominant histological subtype	Superficial	*n* (%)	0 (0.0)
	laBCC	*n* (%)	16 (53.3)		Nodular	*n* (%)	14 (46.7)
	metBCC	*n* (%)	2 (6.7)		Infiltrative	*n* (%)	15 (50.0)
Best response	CR	*n* (%)	6 (22.2)		Sclerosing	*n* (%)	1 (3.3)
	PR	*n* (%)	18 (66.7)	Dominant tumor nest polarity	Palisading	*n* (%)	16 (53.3)
	SD	*n* (%)	2 (7.4)		None	*n* (%)	12 (40.0)
	PD	*n* (%)	1 (3.7)		Front-to-back	*n* (%)	2 (6.7)
Progression under HHI	Yes	*n* (%)	20 (74.1)	Dominant cleft	No cleft	*n* (%)	23 (76.7)
	No	*n* (%)	7 (25.9)		Peritumoral	*n* (%)	7 (23.3)
HHI specific PFS	Median, months (range)		23.4 (4.0–60.9)	Dominant stroma reaction	Loose	*n* (%)	13 (43.3)
					Condensed	*n* (%)	17 (56.7)
	IQR		12.0–39.0	Immune cell infiltrate	Low	*n* (%)	17 (56.7)
Treatment duration	Median. months (range)		10.7 (0.9–109.6)		Intermediate	*n* (%)	10 (33.3)
					High	*n* (%)	3 (10.0)
	IQR		5.1–23.1	Alcian blue distribution	Narrow border	*n* (%)	11 (36.7)
Reason for treatment discontinuation	Progression	*n* (%)	9 (33.3)		Wide border	*n* (%)	6 (20.0)
	AE	*n* (%)	13 (48.2)		Diffuse	*n* (%)	13 (43.4)
	Ongoing	*n* (%)	3 (11.1)	Alcian blue border width	Median, µm (range)		13.8 (2.0–56.9)
	Other	*n* (%)	2 (7.4)				
Follow-up time from HHI initiation	Median, months (range)		36.6 (2.2–117.7)		IQR		10.1–24.6
				Alcian blue pos. single stroma cells	Yes	*n* (%)	13 (43.3)
	IQR		27.0–53.1		No	*n* (%)	17 (56.7)

HHI – hedgehog inhibition; IQR – interquartile range; multiBCC – multiple BCCs; laBCC – locally advanced BCC; metBCC – metastasized BCC; CR – complete response; PR – partial response; SD – stable disease; PD – progressive disease; PFS – progression-free survival; AE – adverse events.

Table S3. Analyzed histological tumor parameters according to clinical BCC stage [European consensus guidelines (EADO)]

Clinical BCC stage			All	Common BCC (st. I/IIA)	MultiBCC (st. IIB)	laBCC (st. III)	metBCC (st. IV)	*p*
n (samples)			70	27	13	25	5	
Dominant histological subtype	Superficial	*n* (%)	11 (15.7)	10 (37.0)	0 (0.0)	1 (4.0)	0 (0.0)	0.003[a]
	Nodular	*n* (%)	34 (48.6)	14 (51.9)	7 (53.8)	11 (44.0)	2 (40.0)	
	Infiltrative	*n* (%)	22 (31.4)	3 (11.1)	6 (46.2)	10 (40.0)	3 (60.0)	
	Sclerosing	*n* (%)	3 (4.3)	0 (0.0)	0 (0.0)	3 (12.0)	0 (0.0)	
Dominant tumor nest polarity	Palisading	*n* (%)	48 (68.6)	25 (92.6)	9 (69.2)	12 (48.0)	2 (40.0)	0.015[a]
	None	*n* (%)	16 (22.9)	1 (3.7)	4 (30.8)	9 (36.0)	2 (40.0)	
	Front-to-back	*n* (%)	6 (8.6)	1 (3.7)	0 (0.0)	4 (16.0)	1 (20.0)	
Dominant cleft	No cleft	*n* (%)	54 (77.1)	22 (81.5)	10 (76.9)	17 (68.0)	5 (100.0)	0.399[a]
	Peritumoral	*n* (%)	16 (22.9)	5 (18.5)	3 (23.1)	8 (32.0)	0 (0.0)	
Dominant stroma reaction	Loose	*n* (%)	37 (52.9)	19 (70.4)	3 (23.1)	12 (48.0)	3 (60.0)	0.040[a]
	Condensed	*n* (%)	33 (47.1)	8 (29.6)	10 (76.9)	13 (52.0)	2 (40.0)	
Immune cell infiltrate	Low	*n* (%)	32 (45.7)	6 (22.2)	6 (46.2)	15 (60.0)	5 (100.0)	0.004[a]
	Intermediate	*n* (%)	23 (32.9)	10 (37.0)	4 (30.8)	9 (36.0)	0 (0.0)	
	High	*n* (%)	15 (21.4)	11 (40.7)	3 (23.1)	1 (4.0)	0 (0.0)	
Alcian blue distribution	Narrow border	*n* (%)	26 (37.1)	11 (40.7)	7 (53.8)	6 (24.0)	2 (40.0)	0.013[a]
	Wide border	*n* (%)	25 (35.7)	14 (51.9)	4 (30.8)	6 (24.0)	1 (20.0)	
	Diffuse	*n* (%)	19 (27.1)	2 (7.4)	2 (15.4)	13 (52.0)	2 (40.0)	
Alcian blue border width	Median, µm (range)		16.2 (2.0–119.8)	17.8 (6.6–59.0)	13.8 (4.5–54.1)	18.2 (2.0–119.8)	15.4 (10.6–37.3)	0.969[b]
	IQR		10.3–29.6	12.8–27.6	11.6–26.9	8.1–29.4	13.0–26.4	
Alcian blue pos. single stroma cells	Yes	*n* (%)	31 (44.3)	16 (59.3)	7 (53.8)	8 (32.0)	0 (0.0)	0.038[a]
	No	*n* (%)	39 (55.7)	11 (40.7)	6 (46.2)	17 (68.0)	5 (100.0)	

[a]Pearson's chi-squared test; [b]Kruskal–Wallis rank sum test.

BCC – basal cell carcinoma; multiBCC – multiple BCCs; laBCC – locally advanced BCC; metBCC – metastasized BCC; IQR – interquartile range.

Table S4. Univariate Cox proportional regression (progression-free survival following HHI treatment initiation). A hazard ratio (HR) >1 indicates a higher risk of progression

Clinical parameter	Category	HR (range)	*p*
Sex	Female (*n* = 15)	Reference	
	Male (*n* = 15)	0.95 (0.39–2.3)	0.901
Age at 1st line treatment > median	No (*n* = 15)	Reference	
	Yes (*n* = 15)	3.4 (1.3–8.7)	0.013*
Gorlin-Goltz-syndrome	Yes (*n* = 7)	Reference	
	No (*n* = 23)	3.7 (1–13)	0.043*
Previous BCC (*n* (NA) = 4)	No (*n* = 7)	Reference	
	Yes (*n* = 19)	0.89 (0.31–2.6)	0.837
Metastasis	No (*n* = 28)	Reference	
	Yes (*n* = 2)	1.7 (0.38–7.5)	0.489
**Conventional tumor parameter**	**Category**	**HR (range)**	***p***
Tumor localization	Body (*n* = 3)	Reference	
	Head (*n* = 27)	0.72 (0.21–2.5)	0.61
Tumor thickness >2 mm (*n* (NA) = 12)	No (*n* = 9)	Reference	
	Yes (*n* = 9)	1 (0.27–3.9)	0.96
Tumor type	Primary (*n* = 18)	Reference	
	Relapse (*n* = 12)	2.2 (0.9–5.3)	0.086
Ulceration (*n* (NA) = 2)	No (*n* = 13)	Reference	
	Yes (*n* = 15)	0.86 (0.34–2.1)	0.742
**Analyzed histological parameter**	**Category**	**HR (range)**	***p***
Histological subtype	Aggressive (*n* = 16)	Reference	
	Non-aggressive (*n* = 14)	0.47 (0.19–1.2)	0.103
Tumor nest polarity	Front-to-back (*n* = 2)	Reference	
	No polarity (*n* = 12)	0.55 (0.115–2.6)	0.448
	Palisading (*n* = 16)	0.37 (0.079–1.7)	0.208
Cleft formation	None (*n* = 23)	Reference	
	Peritumoral (*n* = 7)	0.72 (0.24–2.2)	0.554
Immune cell infiltrate	High (*n* = 3)	Reference	
	Intermediate (*n* = 10)	3.4 (0.39–30)	0.269
	Low (*n* = 17)	6.0 (0.78–47)	0.084
Stroma reaction	Condensed (*n* = 17)	Reference	
	Loose (*n* = 13)	1 (0.44–2.5)	0.915
Alcian blue distribution	Narrow border (*n* = 11)	Reference	
	Wide border (*n* = 6)	1.3 (0.31–5.3)	0.726
	Stroma (*n* = 13)	4.9 (1.63–14.6)	0.005**
Alcian blue single stroma cells	No (*n* = 17)	Reference	
	Yes (*n* = 13)	0.94 (0.39–2.3)	0.885

HR – hazard ratio; BCC – basal cell carcinoma; NA – not available.
